# Comparison of Clinical Efficacy and Anatomical Investigation between Retrolaminar Block and Erector Spinae Plane Block

**DOI:** 10.1155/2019/2578396

**Published:** 2019-03-28

**Authors:** Eiko Onishi, Noriko Toda, Yoshinobu Kameyama, Masanori Yamauchi

**Affiliations:** Department of Anesthesiology, Tohoku University Hospital, Sendai, Miyagi, Japan

## Abstract

Retrolaminar block (RLB) and erector spinae plane block (ESPB) are alternative approaches to paravertebral block (PVB) and are advantageous in that they are easier and safer techniques compared with the traditional PVB. Many clinical reports of these blocks have described their efficacy for ipsilateral thoracic analgesia. The local anesthetic injection points of RLB and ESPB are the lamina and transverse process, respectively. Despite the similarity of the puncture sites, there have been no clinical studies comparing RLB and ESPB. In addition, the underlying mechanism of these blocks has not been clarified. Recent anatomical investigations indicated that the injectate was distributed in the paravertebral space and spread laterally into the intercostal spaces. The limited distribution into the paravertebral space indicated that compared to PVB, RLB and ESPB exert their effects via a different mechanism. In this review, we describe the features of and differences between RLB and ESPB based on current clinical and anatomical reports. We also propose the clinical indication and discuss the differences, clinical outcomes, and anatomical mechanisms of the techniques.

## 1. Introduction

For an ideal perioperative regional anesthetic technique, most anesthesiologists would prefer a safe, easy, and minimally invasive procedure that can be performed in a shorter time frame and provide appropriate analgesia. The development of ultrasonography led to the establishment of ultrasound (US-) guided nerve blocks. US-guided nerve blocks are now commonly used as a part of the multimodal postoperative analgesic strategy. Various approaches to US-guided peripheral nerve block (PNB) have been reported recently [1–6], including intramuscular, compartment, and interfascial plane blocks. The site of injection is not the perineural space but the space through which the peripheral branch of the target nerve runs.

**Figure 1:**
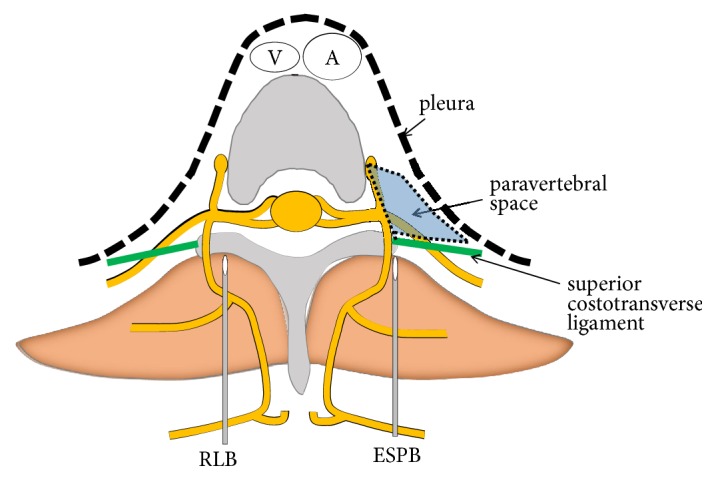
*The injection point of the retrolaminar block and erector spinae plane block*. The needle used for retrolaminar block (RLB) is inserted 1 cm lateral to the spinous process and local anesthetic is injected on the lamina. The needle used for erector spinae plane block (ESPB) is inserted 2-3 cm lateral to the spinous process and local anesthetic is injected on the transversus process. In both RLB and ESPB, the needle is not required to penetrate the superior costotransverse ligament.

Thoracic epidural anesthesia (TEA) and paravertebral block (PVB) have been used to provide perioperative regional anesthesia in the trunk. However, TEA is technically difficult in some cases, and is associated with a risk of serious complications, such as epidural hematoma, nerve injury, and hypotension. PVB has the advantage of visualization of the needle position using ultrasonography. However, PVB is also associated with a risk of serious complications, such as pneumothorax, hypotension, or nerve injury. Newer approaches to PVB have been the focus of many studies in recent years; these approaches include retrolaminar block (RLB) [5] and erector spinae plane block (ESPB) [6]. These blocks are considered to be compartment blocks or interfascial plane blocks. In these approaches, local anesthetics are assumed to penetrate the superior costotransverse ligament and reach the paravertebral space, although the needle tip is not advanced into the paravertebral space (Figure 1). The clinical effect of RLB and ESPB has been reported for ipsilateral thoracic surgery. Further, considering the close puncture sites of RLB and ESPB (Figure 1), the similarities between RLB and ESPB have been discussed previously [7, 8]. However, the injectate distribution patterns and the mechanisms of spinal nerve blockade of both techniques remain unclear. Anatomical evidence may be crucial to aid our understanding of the nature of these blocks.

In this review, we demonstrate the features and differences with regard to the clinical efficacy and anatomical distribution of RLB and ESPB. We also aim to clarify the appropriate indications for RLB and ESPB.

## 2. RLB and ESPB Techniques

RLB was first reported in 2006 as an alternative approach to PVB [9]. RLB is performed with US imaging or the landmark technique. The needle is inserted at a puncture site 1-1.5 cm lateral to the target spinous process and advanced caudally or cranially until it contacts the lamina (Figure 1). Local anesthetics are injected on the lamina at doses of 20-30 ml.

ESPB was first reported in 2016 for ipsilateral thoracic analgesia [6]. The needle is inserted at a puncture site 2-3 cm lateral to the target spinous process using US imaging and advanced until contact is made with the transverse process (Figure 1). The needle-tip in ESPB is advanced to a more superficial point than that in PVB; thus, visualization of the needle using ultrasonography is considered to be easier in ESPB than in PVB. The local anesthetic (20-30 ml) is injected between the transverse process and the erector spinae muscle.

**Figure 2:**
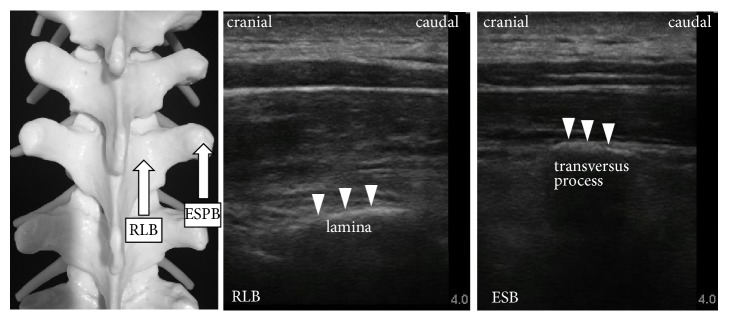
*Ultrasound images of retrolaminar block and erector spinae plane block*. The sagittal plane with a linear ultrasound probe allows for visualization of the laminae or transversus process. The insertion points for retrolaminar block (RLB) and erector spinae plane block (ESPB) are similar. The transversus process is more superficial than the lamina in the ultrasound image, and the injection point for ESPB is close to the pleura.

These two compartment blocks can be performed with the US-guided, in-plane insertion technique. The sagittal plane with a linear US probe allows for visualization of the laminae or transversus process (Figure 2), and the needle is advanced using the in-plane technique. With regard to complications, only one case report of pneumothorax after ESPB has been reported [10]. ESPB cannot be performed with the landmark technique, because the transverse process is not detected by palpation. Hence, using ultrasonography is essential in ESPB. However, RLB can be performed with the landmark technique. The needle can be advanced to the lamina and the target spinous process can be palpated, similar to the paramedian approach in thoracic epidural puncture. The technique of RLB is simpler and easier than that of ESPB. We also previously recommended that the RLB needle be advanced perpendicular to the skin or cephalad to caudal in order to avoid epidural injection [11]. No complications of RLB have been reported.

## 3. Comparison of RLB and ESPB with PVB and TEA

The technical features of RLB, ESPB, PVB, and TEA are summarized in Table 1. TEA is the most common technique and provides both somatic and visceral analgesia. However, the failure rate is reported to be 14-30% [12]; significant skill and experience are needed to perform TEA. The complications associated with TEA are accidental dural puncture, hypotension, spinal injection, nerve injury, and epidural hematoma [12]. In contrast, US-guided PVB was reported to be associated with very few complications [13]. However, the needle-tip must be close to the pleura and spinal nerve roots. PVB has been classified as a technique of advanced level of difficulty [14].

The advantage of RLB and ESPB is that they are technically easier procedures than PVB and TEA. The needle-tip of RLB and ESPB is not closer to pleura and spinal nerve roots than that of TEA and PVB. Furthermore, using US images allows for visualization of the needle and local anesthetic distribution. However, the available information regarding these blocks is not sufficient; the optimal dose of local anesthetics, area of sensory block, and differences between single and multilevel injection or single injection and continuous injection remain to be clarified.

## 4. Clinical Reports

Clinical case reports on both RLB and ESPB have demonstrated the efficacy of these techniques. However, only a few randomized controlled trials (RCTs) have investigated RLB and ESPB.

The efficacy of continuous RLB has been reported for breast cancer surgery [15, 16] and rib fracture [5, 17]. These reports of successful cases indicated RLB to be an effective method as an alternative to PVB or multiple intercostal nerve blocks. However, some reports questioned whether RLB offers an analgesic effect equivalent to that of PVB. Satoh demonstrated that the mixtures of local anesthetics and contrast dye were distributed across the laminae cephalocaudally and did not disperse into the paravertebral space in a radiographic study [18]. Additionally, Murouchi et al. evaluated the use of continuous RLB for breast cancer surgery as compared with PVB [19]. They reported that the analgesic effect of RLB was weaker than that of classic PVB. We previously reported an RCT of single-shot RLB for breast cancer surgery [11], which is the only RCT of RLB. We found that the use of RLB did not reduce the number of patients requiring postoperative analgesia, and the postoperative analgesic duration was only 2-3 h, which was unexpectedly shorter than that of PVB reported previously. In our study, we performed RLB without ultrasonography and injected a lower volume of local anesthetics at two sites: 15 ml at T2 and T4, respectively. These methods could potentially have influenced our results.

However, the efficacy of ESPB has been described in a greater number of clinical reports than has RLB: a rib fracture [20], breast surgery [21, 22], thoracoscopic surgery [23, 24], lumbar spinal surgery [25, 26], and laparoscopic abdominal surgery [27, 28]. In contrast to RLB, the majority of the literature on ESPB reported the use of the single-shot technique (80.2%) [29]. The local anesthetic was postulated to infiltrate the ventral and dorsal rami of the spinal nerve. However, Ueshima et al. reported that ESPB could not provide adequate analgesia of the anterior branch of the intercostal nerve [30]. Therefore, the mechanism of ESPB as a PVB is controversial.

In 2018, three RCTs of ESPB were reported. Tulgar et al. evaluated the postoperative analgesia provided by ESPB in laparoscopic cholecystectomy [28]. They demonstrated that bilateral single-shot ESPB performed before general anesthesia induction significantly reduced postoperative pain in the initial 3 h and the requirement for postoperative analgesia in the initial 24 h compared with the general anesthesia alone technique. Gürkan et al. also demonstrated that the preoperative single-shot ESPB reduced the postoperative morphine consumption within 24 h after surgery in patients undergoing breast cancer surgery [21]. Additionally, Krishna et al. demonstrated that the bilateral ESPB for cardiac surgery provided significantly superior analgesia in the acute postsurgical phase and a longer duration of analgesia than that provided in the control group [31]. Although these RCTs were single-blinded studies, they demonstrated the clinical efficacy of single-shot ESPB.

The clinical case reports on RLB and ESPB indicated their potential as alternative methods of PVB and TEA. The RCTs reported that the clinical efficacy of single-shot RLB was controversial, while ESPB resulted in significantly lower postoperative pain. However, no reports have compared RLB and ESPB or investigated the optimal dose and concentration of local anesthetics. High quality randomized trials are needed to evaluate clinical efficacy of RLB and ESPB.

## 5. Anatomical Studies

In 2016, Costache et al. reported that the superior costotransverse ligament was not a barrier to the diffusion of the injectate, and local anesthetics could penetrate the ligament toward the paravertebral space [32]. This report supported the hypothesis that the local anesthetics injected in RLB or ESPB would penetrate the paravertebral space. Subsequently, in 2018, several cadaveric anatomical investigations were reported, which provided meaningful results for the clarification of the mechanisms of RLB and ESPB [33–35]. The anatomical investigations are summarized in Table 2.

Initially, Ivanusic et al. performed a cadaveric experiment to determine whether the injectate of ESPB dispersed anteriorly into the paravertebral space [33]. They injected 20 ml of 0.25% methylene blue dye into the plane between the fifth thoracic transverse process and the erector spinae muscle of unembalmed cadavers. They demonstrated that the injectate was distributed craniocaudally and laterally along the erector spinae muscles, and the dorsal ramus was stained with dye. However, the paravertebral space and intercostal nerves were not stained with dye. They also demonstrated that the extensive lateral diffusion of local anesthetics could involve the lateral cutaneous branches of the intercostal nerves, which allowed wide cutaneous sensory block of the hemi-thorax.

In contrast, Adhikary et al. performed a comparative study of the distribution of 20 ml of injectate of RLB and ESPB in fresh cadavers, using both magnetic resonance imaging and anatomical dissection [34]. Single-shot RLB and ESPB both produced epidural and neural foraminal diffusion across two to five vertebral levels centered around the level of injection, which indicated that both techniques elicit clinical effects similar to those of PVB. In particular, the injectate of ESPB was dispersed more widely into the intercostal space than was RLB. ESPB could provide analgesia of the anterolateral thoracic and abdominal wall as an intercostal nerve block.

Sabouri et al. also investigated the distribution of local anesthetic after RLB using unembalmed, fresh frozen, and thawed cadavers [35] using injection of 20 ml of a mixture of 1 ml of 1% methylene blue and 19 ml of 0.5% bupivacaine. They demonstrated that the injectate of the retrolaminar space could diffuse into the paravertebral space, epidural space, and intervertebral foramina. However, the pattern of injectate dispersion in RLB was variable, and the diffusion into the paravertebral space might be more limited than that of classic PVB.

Additionally, Yang et al. demonstrated that the injectates of RLB and ESPB reached the paravertebral space and infiltrated the superior costotransverse ligament in unembalmed cadavers [36]. The area stained with dye in ESPB was more lateral, whereas the dye spread vertically along the posterior surface of the lamina in RLB. These findings indicated that RLB could involve the dorsal rami of the spinal nerve and may be more suitable for the analgesia of the thoracic back region than is ESPB.

In these anatomical investigations, the difference in the tissue condition between a cadaver and a living person was described as a limitation. The possibility that tissue manipulation during dissection could have caused dye dispersion to deeper areas such as the epidural space and paravertebral space was also not excluded. Thus, the anatomical mechanisms of RLB and ESPB have not been fully clarified. The areas of injectate distribution in both RLB and ESPB differed among studies. However, the patterns of injectate distribution can be summarized according to the following three points. First, the dye in RLB was distributed vertically beneath the transversospinalis muscles, and the dorsal rami of spinal nerve could be blocked. Second, the dye in ESPB spread laterally, and the intercostal nerve or the lateral cutaneous branches of intercostal nerve could be blocked. Finally, the distribution into the paravertebral space was limited in both RLB and ESPB.

## 6. Anatomical Mechanism as an Interfascial Plane Block

**Figure 3:**
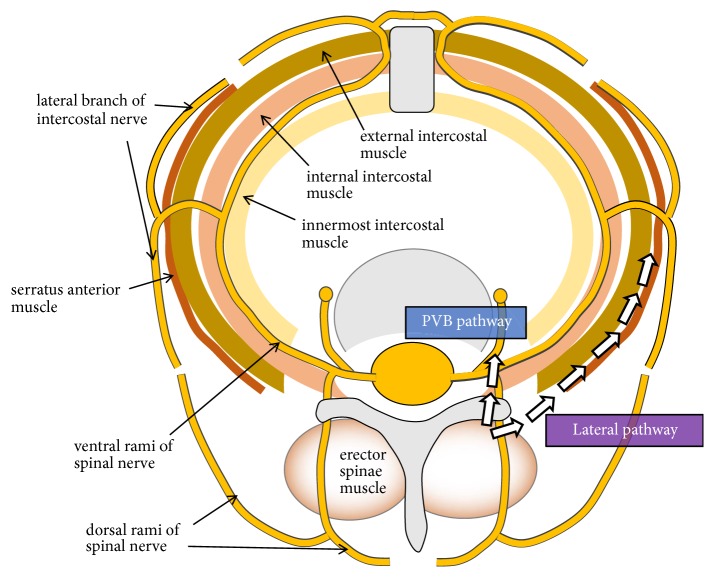
*The distribution pathway of local anesthetics in retrolaminar block and erector spinae plane block*. The paravertebral block (PVB) pathway involves both the ventral and the dorsal spinal rami and showed similar mechanism with PVB. The lateral pathway involves the lateral cutaneous branch and small branches of intercostal nerves. This pathway was similar to the mechanism underlying interfascial plane block.

Elsharkawy et al. reported very interesting findings regarding the interfascial plane block [37]. They described that the retrolaminar space and erector spinae muscle plane are directly linked to the interfascial plane, through which the lateral cutaneous branch runs. If the local anesthetics injected in RLB or ESPB are distributed in the interfascial plane, the blockade of the lateral cutaneous branch can provide hemithoracic analgesia. This report suggested that RLB and ESPB would have a mechanism as an interfascial plane block. Ivanusic et al. also visualized the small branches of the intercostal nerves running through the external intercostal muscle layer in an anatomical investigation [38]. If the local anesthetics injected via RLB or ESPB were laterally distributed as an interfascial plane block in a live body, these small branches would be infiltrated, which could provide hemithoracic analgesia (Figure 3).

Summarizing these anatomical investigations and literature regarding interfascial plane block, RLB and ESPB could have two mechanisms; “the PVB pathway” and “the lateral pathway.” Firstly, the PVB pathway involves both ventral and dorsal spinal rami. However, the limited distribution of local anesthetics can cause the potency of RLB and ESPB to be weaker than that of PVB. Secondly, the lateral pathway involves the lateral cutaneous branch and small branches of intercostal nerves. The blockade of these cutaneous branches of intercostal nerves can provide a certain level of analgesia for the hemithorax. The injectate of ESPB is distributed deeper and more lateral than that of RLB. Therefore, ESPB is likely to produce larger area of anesthesia than that of RLB.

## 7. Future Investigations and Clinical Indications

Anatomical studies have demonstrated restricted distribution of dyes in both RLB and ESPB in the paravertebral space, which indicates that the analgesic adequacy of the two blocks might be less certain than that of PVB and TEA. The optimal dose and volume of local anesthetics for RLB and ESPB have not been elucidated. A recent anatomical study of RLB using porcine cadavers suggested that the dye was distributed to the paravertebral space in a volume-dependent manner [39]. De Cassai et al. also evaluated the volume of local anesthetics in ESPB. They demonstrated that a median volume of 3.4* *ml of local anesthetic was required to anesthetize one dermatome. The volume of local anesthetics will be an important factor in determining the anesthetized area for both RLB and ESPB. Further investigation regarding the optimal dose and volume of local anesthetics and the sensory block area is necessary to establish the techniques of RLB and ESPB.

In anatomical studies, the dye injected via an RLB tends to infiltrate the dorsal rami of the spinal nerve, and the lateral pathway is not involved in the main mechanism of RLB. Technically, RLB is easy and safe and can be performed without ultrasonography. RLB will be suitable for ambulatory patients or patients with back pain. In contrast, the lateral pathway is the main mechanism of ESPB. The blockade of the intercostal nerve or the lateral cutaneous branches of the intercostal nerves would provide hemithoracic analgesia. Therefore, ESPB will be useful for perioperative analgesia in hemithoracic surgery. However, the PVB pathway of RLB and ESPB is varied and restricted; hence, these blocks could not be a solo anesthesia technique for thoracic wall surgery. TEA and PVB should also be performed for supplemental analgesia, if adequate analgesia cannot be achieved using these two blocks.

## 8. Conclusion

We reviewed the clinical features and anatomical findings of RLB and ESPB. The advantages of these techniques are that they are easy and have low risks of severe complications. The anatomical investigations demonstrated that the distribution of the injectate in RLB and ESPB follows the PVB pathway and the lateral pathway. However, the mechanisms of RLB and ESPB have not been fully clarified. The clinical efficacy presented in reports including case reports and control studies suggests that these blocks may be potentially useful for anesthesiologists. However, only a few RCTs of RLB and ESPB have been reported. To establish RLB and ESPB as routine procedures in the clinical setting, high-quality RCTs will be essential in the future.

## Figures and Tables

**Table 1:** The features of RLB, ESPB, PVB, and TEA.

	RLB	ESPB	PVB	TEA
Difficulty	probably easy		advanced level	

Technique				
Methods	landmark or ultrasound	ultrasound	ultrasound	landmark
Needle-tip position	lamina	transversus process	paravertebral space	epidural space

Laterality of effects	unilateral	unilateral	unilateral	bilateral

Complications	rare	rare	hypotension	epidural hematoma
			(less than TEA)	epidural abscess
			pneumothorax	paralysis
			epidural injection	nerve injury
			hematoma	hypotension
				dural puncture

RLB: retrolaminar block, ESPB: erector spinae plane block, PVB: paravertebral block, and TEA: thoracic epidural anesthesia.

**Table 2:** The comparison of anatomical investigations.

			Ivanusic et al.	Adhikary et al.	Sabouri et al.	Yang et al.
Target of the investigation			ESPB	RLB and ESPB	RLB	RLB and ESPB

Method			20 injection in 10 cadavers	3 injection per each block	8 injection in 8 cadavers	10 injection per each block

Cadaver			unembalmed/fresh cadavers			

Injection level			T5 vertebral level	T5 vertebral level	T4 vertebral level	T5 vertebral level

Injectate volume			20 ml of mixture			

The spread of dye	Anterior	Paravertebral space	none	possible in both blocks	possible (quite variable)	limited spread
		Epidural space				no details
		Intercostal space				no details
	Posterior		The dorsal ramus was frequently stained.	RLB: beneath the transversospinalis muscles ESPB: beneath the erector spinae muscles	The dye spread beneath the paraspinal muscles.	RLB: the surface of the transversospinalis muscle ESPB: the surface of the external intercostal muscle
	Lateral		The dye often spread to the attachments of iliocostalis muscle.	ESPB > RLB RLB: to the edge of the bony lamina ESPB: extending 9 to 10 cm lateral	median lateral spread 2.5cm	RLB < ESPB RLB: limited medially to the intertransverse ligaments ESPB: spread to iliocostalis muscle
	Longitudinal		The majority of the dye spread was cephalad to T6.	ESPB > RLB RLB: 6-9 vertebral levels ESPB: 9-14 vertebral levels	median cephalad spread 3.5cm caudad spread10.7cm	spinal nerve involvement: RLB (2 levels) > ESPB (3.5 levels)

RLB: retrolaminar block and ESPB: erector spinae plane block.
